# PfsR Is a Key Regulator of Iron Homeostasis in *Synechocystis* PCC 6803

**DOI:** 10.1371/journal.pone.0101743

**Published:** 2014-07-10

**Authors:** Dan Cheng, Qingfang He

**Affiliations:** Department of Applied Science, University of Arkansas at Little Rock, Little Rock, Arkansas, United States of America; Universidade Federal de Vicosa, Brazil

## Abstract

Iron is an essential cofactor in numerous cellular processes. The iron deficiency in the oceans affects the primary productivity of phytoplankton including cyanobacteria. In this study, we examined the function of PfsR, a TetR family transcriptional regulator, in iron homeostasis of the cyanobacterium *Synechocystis* PCC 6803. Compared with the wild type, the *pfsR* deletion mutant displayed stronger tolerance to iron limitation and accumulated significantly more chlorophyll a, carotenoid, and phycocyanin under iron-limiting conditions. The mutant also maintained more photosystem I and photosystem II complexes than the wild type after iron deprivation. In addition, the activities of photosystem I and photosystem II were much higher in *pfsR* deletion mutant than in wild-type cells under iron-limiting conditions. The transcripts of *pfsR* were enhanced by iron limitation and inactivation of the gene affected pronouncedly expression of *fut* genes (encoding a ferric iron transporter), *feoB* (encoding a ferrous iron transporter), *bfr* genes (encoding bacterioferritins), *ho* genes (encoding heme oxygenases), *isiA* (encoding a chlorophyll-binding protein), and *furA* (encoding a ferric uptake regulator). The iron quota in *pfsR* deletion mutant cells was higher than in wild-type cells both before and after exposure to iron limitation. Electrophoretic mobility shift assays showed that PfsR bound to its own promoter and thereby auto-regulated its own expression. These data suggest that PfsR is a critical regulator of iron homeostasis.

## Introduction

Iron is an essential cofactor in many critical cellular processes, including photosynthesis, respiration, nitrogen fixation, chromophore biosynthesis, and gene regulation. Iron has been considered as a limiting nutrient affecting the marine primary production because iron is poorly soluble in oxygenated seawater [1], [2].

The photosynthetic cyanobacteria comprise a large fraction of marine phytoplankton and adapt to the iron-limiting ecosystems, which makes them excellent models for studying iron stress tolerance in relation to photosynthesis. Due to the importance of iron in the photosynthetic electron transport chain, the iron quota in cyanobacteria is much higher than in non-photosynthetic bacteria [3]. The photosynthetic apparatus of cyanobacteria contains a substantial amount of iron in the form of heme, non-heme iron, and iron-sulfur clusters [4]. Photosystem II (PSII) contains three iron atoms; the cytochrome b_6_f complex has five; photosystem I (PSI), which is the largest pool of iron in photosynthetic systems, contains 12 iron atoms; and ferredoxin, the terminal electron acceptor, has two iron atoms [5]. During iron limitation, cyanobacteria synthesize less iron-containing components of the photosynthetic electron transport chain, especially the most iron-expensive complex PSI, leading to a significant decrease in PSI∶PSII ratio [6]–[10]. Therefore, the availability of iron may determine the structure and function of the photosynthetic apparatus and affect the primary productivity of cyanobacteria.

Cyanobacteria also exhibit various other physiological and morphological changes in response to iron limitation. For instance, iron deficiency causes a decrease in cellular pigmentation (such as chlorophyll a and phycocyanin) [6], results in a blue shift in the main red absorption band of chlorophyll a [11], activates iron acquisition mechanisms [12], leads to the replacement of iron-containing enzymes with functionally similar iron-free counterparts (such as ferredoxin with flavodoxin) [13], and, most strikingly, up-regulates the expression of IsiA, a chlorophyll-binding protein unique to cyanobacteria [14]–[16]. Under iron-limiting conditions, IsiA accumulates in the thylakoid membrane, where it binds to chlorophyll and forms IsiA-PSI supercomplexes consisting of a PSI trimer encircled by 18 IsiA monomers [9], [15], [17]–[19]. IsiA may also form supercomplexes with PSI monomers or even without PSI [20], [21]. More recent studies have reported that IsiA could form two rings around a PSI trimer or form IsiA-PSI-PSII supercomplexes in the iron-limited cyanobacterial cells [22], [23]. The IsiA supercomplexes are thought to increase the light capture ability of PSI and protect the iron-limited cells from oxidative damage [9], [24]–[29]. Furthermore, it has been found that IsiA is important for efficient state transition in *Synechocystis* PCC 6803 (hereafter referred to as *Synechocystis* 6803) cells [22].

Here, we describe a unique cyanobacterial mutant, *pfsR* (photosynthesis, Fe homeostasis and stress-response regulator) deletion mutant, that exhibits enhanced tolerance to iron limitation. PfsR, which is encoded by the open reading frame (ORF) *sll1392* in *Synechocystis* 6803, was discovered in a genetic suppressor screen [30]. Inactivation of PfsR suppressed the lethality of high light to the high light-sensitive strain lacking four *hli* genes, due to the tighter control of free intracellular iron levels [30]. PfsR resembles the tetracycline repressor (TetR) family of transcriptional regulators in terms of protein sequence and predicted secondary structure and contains a TetR-HTH (helix-turn-helix) motif at its N-terminus. TetR regulators are the third most common family of transcriptional regulators in bacteria [31], [32] and are believed to facilitate adaptation to complex environments. Most TetR family regulators function as repressors by binding to target operators [31].

In this study, we investigated the regulatory role of PfsR in iron homeostasis. Inactivation of PfsR in *Synechocystis* 6803 enhanced its viability and photosynthetic activity under iron-limiting conditions. Additionally, we demonstrated that PfsR was auto-regulated and modulated iron homeostasis by regulating a set of genes related to iron homeostasis, including *fut* genes, *feoB*, *bfr* genes, *ho* genes, *isiA*, and *furA*.

## Materials and Methods

### Growth conditions and iron treatment

*Synechocystis* 6803 strains were grown in BG-11 medium at 30°C under 50 µmol photons m^−2^s^−1^ continuous white light and bubbling with air. Cell density was determined by measuring the optical density (OD) of the suspension at 730 nm (OD_730_) with a spectrophotometer (DU-70, Beckman Coulter, Brea, CA). To determine sensitivity to iron, cells grown in BG-11 medium were diluted to OD_730_ about 0.2 and grown in BG-11 medium that lacked ferric ammonium citrate and contained 10 µM 2,2′-dipyridyl, an iron chelator.

### Pigment analysis

The chlorophyll a and total carotenoid in cells were extracted using dimethylformamide (DMF), and their concentrations were determined using the formula [33]:

[Total Carotenoids] (µgml^−1^) = (OD_461_–0.046×OD_664_)×4.

[Chlorophyll a] (µgml^−1^) = 12.1×OD_664_–0.17×OD_625_.

The loss of phycobiliprotein absorbance in samples heated to 75°C was used to determine phycocyanin content according to the following formula [34]:
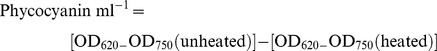



### 77K chlorophyll fluorescence and room-temperature chlorophyll fluorescence analysis

Fluorescence emission spectra at 77K were measured using a Fluormax-4 spectrofluorometer (Horba Jobin YVON, Japan) at an excitation wavelength of 430 nm with excitation bandwidth of 5 nm and emission bandwidth of 1.5 nm. The chlorophyll concentration was adjusted to 15 µgml^−1^.

Chlorophyll fluorescence yield was monitored using a DUAL-PAM-100 P700 & Chlorophyll Fluorescence Measuring System (Heinz Walz, Germany) and DUAL-PAM software. The maximal quantum yield of PSII was determined as (F_m_–F_o_)/F_m_ = F_v_/F_m_. The effective quantum yield of PSII [Y(II)] was determined as (F_m_’–F)/F_m_’ = Y(II). The P700 parameter P_m_, the determination of which involves far-red preillumination and application of a saturation pulse, represents the maximal change of the P700 signal. The fraction of overall P700 that is oxidized in a given state (P700^+^) is calculated as P700^+^ = 1–(P_m_’–P)/P_m_. To determine the light-intensity dependence of relative electron transport rates (ETR), actinic light intensities were increased stepwise from 0 to 1500 µmol of photons m^−2^s^−1^.

### Oxygen evolution

Cells were grown to the exponential phase and harvested by centrifugation at 500×g for 5 minutes. Whole-chain-, PSII-, and PSI-mediated oxygen evolution activities were measured using a Clark-type electrode at light intensity of 1000 µmol of photon m^−2^s^−1^. Intact cells containing 5 µgml^−1^ of chlorophyll were used in the measurement of whole-chain and PSII-mediated electron transport rate, and thylakoid membranes containing 15 µgml^−1^ of chlorophyll were used to measure PSI-mediated electron transport rate. In order to measure electron transport rate of whole-chain, 1 mM NaHCO_3_ was added to cell cultures. The PSII–mediated electron transport (from water via PSII to dichloro-p-benzoquinone) rate was measured in the presence of 1 mM K_3_FeCN and 0.5 mM 2,6-dichloro-p-benzoquinone. The PSI-mediated electron transport (from DCIP/ascorbic acid via PSI to MV) rate was measured in thylakoid membrane suspensions containing 40 µM methylviologen (MV), 5 mM NH_4_Cl, 2 mM ascorbic acid, 0.1 mM 2,6-dichlorophenolindophenol (DCPIP), 2 mM NaN_3_, 40 µM 3-(3,4-dichlorophenyl)-1,1-dimethylurea (DCMU), 40 mM tricine (pH 7.5), and 100 mM sucrose.

### Immunoblot

Thylakoid membrane isolation was performed according to the previously reported method [35]. Solubilization of thylakoid membranes and sodium dodecyl sulfate-polyacrylamide gel electrophoresis (SDS-PAGE) were performed as described [36].

Approximately 20 µg of thylakoid membrane proteins were resolved by SDS-PAGE in 12% polyacrylamide gels with 6 M urea, followed by transferring to nitrocellulose membranes. Immonodetection of proteins was performed using commercial primary antibodies (Agrisera, Sweden). Bound antibodies were detected using anti-rabbit IgG conjugated to alkaline phosphatase (Bio-Rad, Hercules, CA). Protein bands were visualized using a NBT/BCIP reagent kit (Invitrogen, Carlsbad, CA) and quantified using Image J software (http://imagej.nih.gov/ij/).

### Heme staining

Heme staining was detected by peroxidase activity as described [37]. Briefly, the polyacrylamide gel was immersed in a solution containing 6.3 mM 3,3′,5,5′-tetramethylbenzidine (TMBZ), 250 mM sodium acetate (pH 5.0), and 25% methanol for 60 minutes. The heme was visualized within 30 minutes after the addition of 2% H_2_O_2_. The density of the bands was analyzed using Image J software.

### Quantitative PCR

Relative transcript levels of various genes were evaluated by quantitative PCR. Briefly, total RNA was isolated from 100 ml *Synechocystis* 6803 cultures as described [35], followed by treatment with RNase free-DNase (Qiagen, Germany), and then purified using RNeasy Mini Kit (Qiagen, Germany). Reverse transcription reaction was performed using Superscript III reverse transcriptase (Invitrogen, Carlsbad, CA) and random primers. The resultant cDNA was subjected to quantitative PCR analysis (CFX96, Bio-Rad, Hercules, CA) using specific primers for different genes (Table S1). The 16S rRNA gene was used as an internal control to normalize mRNA abundance. Comparative C_t_ (△△C_t_) method was used to quantify the relative levels of gene expression. Three biological replicates with three PCR replicates each were performed.

### Iron content measurement

Cells were harvested by centrifugation at 500×g for 5 minutes, washed three times with 10 mM EDTA and three times with distilled water, and then dried at 50°C for 48 hours. The dried cells were digested in 70% nitric acid. After all the cells were dissolved, the nitric acid was evaporated and the samples were then reconstituted in 5% nitric acid. Samples were centrifuged at 1100×g for 5 minutes and the supernatant was analyzed for iron content. Atomic absorption was performed in the furnace mode on a Perkin-Elmer Atomic Absorption Spectrometer model AA600 (Perkin-Elmer, Ueberlingen, Germany).

### Heterologous expression and purification of His_6_-tagged PfsR protein

For overexpression of PfsR protein, the full-length *pfsR* ORF was amplified by PCR and cloned into the Sph I and Bgl II sites of the pQE-70 vector (Qiagen, Germany). PfsR expression was induced in *E. coli* cultures with 1 mM isopropyl β-D-thiogalactopyranoside (IPTG). The cells were then grown for an additional 8 hours at 20°C. After harvesting the cultures, the cells were broken using a bead beater. His_6_-tagged PfsR protein was purified under native conditions, using a TALON Spin Column (Clontech Laboratories, Mountain View, CA) according to the manufacturer’s instructions. The expression level and purity of the proteins were tested by SDS-PAGE analysis.

### Electrophoretic mobility shift assay (EMSA)

The binding of PfsR to the promoter regions of a number of genes was determined by EMSA. DNA fragments for EMSA were amplified by PCR from *Synechocystis* 6803 genomic DNA. The primers used are listed in Table S1. Each reaction (20 µl) contained 0.05 µM DNA fragments, 0 to 4 µM of purified PfsR protein, and binding buffer [4 mM Tris-HCl (pH 7.5), 12 mM HEPES (pH 7.5), 50 mM NaCl, 10 mM MgCl_2_, 12% glycerol]. The reactions were incubated at 25°C for 30 minutes and then subjected to 6% native polyacrylamide gel electrophoresis at 200 V in 0.5×Tris-borate-EDTA (TBE) buffer. The binding of PfsR to promoter regions was detected using the Electrophoretic Mobility Shift Assay (EMSA) Kit (Invitrogen, Carlsbad, CA), according to the manufacturer’s instructions.

## Results

### The *pfsR* deletion mutant displays enhanced tolerance to iron limitation

**Figure 1:**
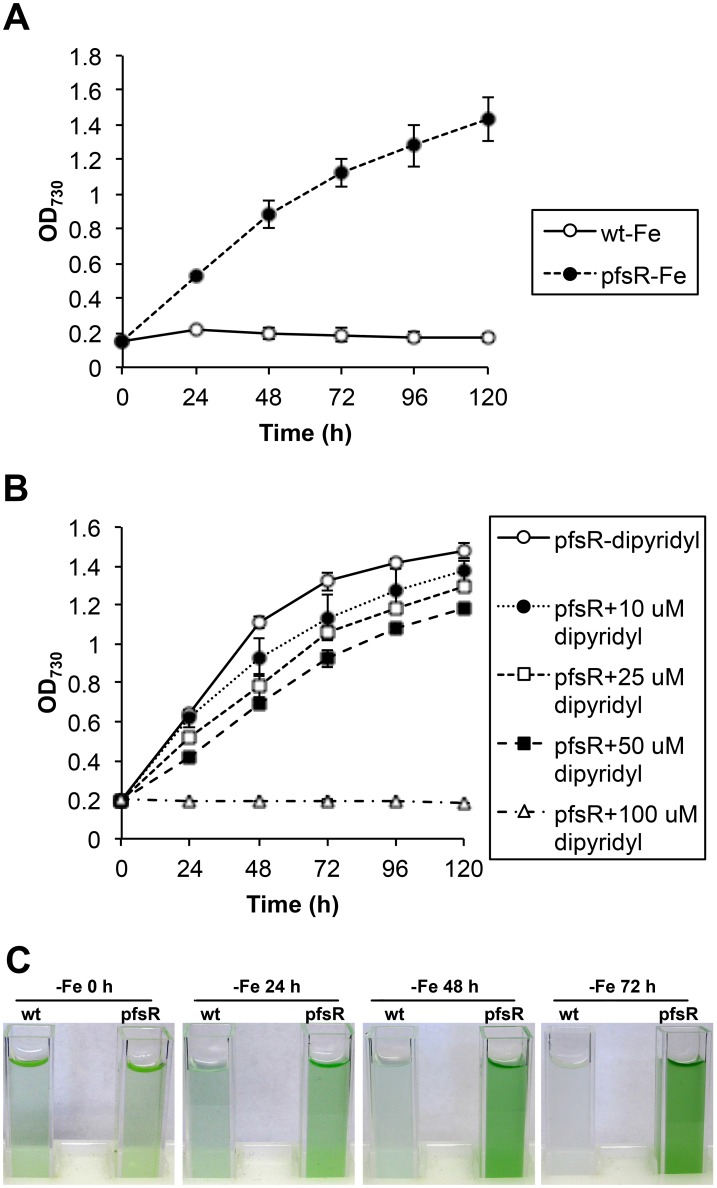
The effect of iron limitation on the growth of wild-type and *pfsR* deletion mutant cells. A, Growth of the wild type (wt, open circles) and the *pfsR* deletion mutant (pfsR, closed circles) under iron-limiting conditions. Growth was determined by measuring the optical density at 730 nm (OD_730_). B, Growth of the *pfsR* deletion mutant in the presence of increasing concentrations (0 µM, open circles; 10 µM, closed circles; 25 µM, open squares; 50 µM, closed squares; 100 µM, open triangles) of the iron chelator 2,2′-dipyridyl. C, Wild-type and *pfsR* deletion mutant cell cultures under iron-limiting conditions. At the exponential growth phase (OD_730_ of 0.6 to 0.8), wild-type and *pfsR* deletion mutant cells were diluted to OD_730_ of 0.2, incubated in iron-free BG-11 medium, and treated with 10 µM (A and C) or various concentrations (B) of 2,2′-dipyridyl (dipyridyl) for the indicated periods of time. The data are means of three biological replicates with error bars indicating the standard deviations (SD).

Previous study has linked PfsR to iron homeostasis [30]. In order to further evaluate the effect of *pfsR* deletion on iron stress responses, we examined cell growth of the wild type and the *pfsR* deletion mutant under conditions with different iron availability. Cells grown in normal BG-11 medium to the exponential phase were washed with iron-free BG-11 medium, and then diluted to an OD_730_ of 0.2 in iron-free BG-11 medium with supplement of 10 µM iron chelator 2,2′-dipyridyl. Under iron-replete conditions, the growth rates of the wild type and the *pfsR* deletion mutant were virtually indistinguishable (data not shown). In contrast, the wild type grew poorly and the *pfsR* deletion mutant grew well under iron-limiting conditions (Fig. 1A and C). The growth rate of the *pfsR* deletion mutant decreased as the concentration of 2,2′-dipyridyl increased, and growth ceased at a concentration of 100 µM (Fig. 1B). These results indicate that the *pfsR* deletion mutant has enhanced viability under iron-limiting conditions compare to the wild type.

The iron-limiting media used in this study included 2,2′-dipyridyl, which is an iron chelator widely applied in cyanobacteria to induce iron stress [30], [38]–[40]. 2,2′-dipyridyl competes for intracellular free iron in addition to sequestering traces of iron that might exist in iron-free medium, thus depletes iron more efficiently and rapidly than the other commonly used iron-deprivation method (i.e. depleting iron by repeated washing and dilution in iron-free medium). Additionally, comparing with other iron chelators, such as deferoxamine B, which was reported to induce mild iron limitation (with unaffected growth) in *Synechocystis* 6803 [3], 2,2′-dipyridyl causes more severe iron limitation, which restricts cell growth within a relatively short period of time (Fig. 1).

### The *pfsR* deletion mutant accumulates more photosynthetic pigments than the wild type under iron-limiting conditions

**Figure 2:**
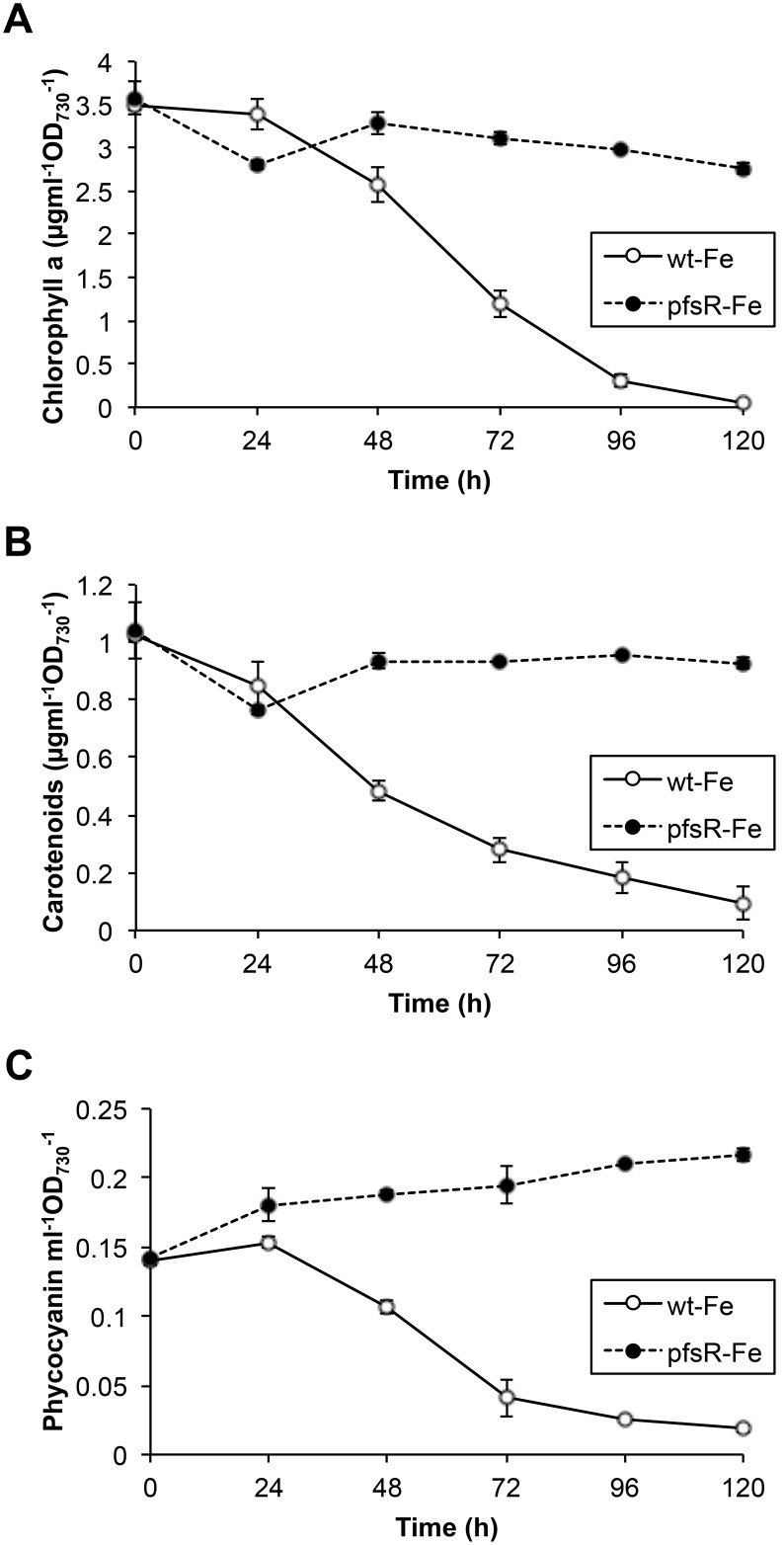
Photosynthetic pigment accumulation under iron-limiting conditions. Pigment levels were measured in the wild type (open circles) and the *pfsR* deletion mutant (closed circles) grown under iron-limiting conditions for various lengths of time. Chlorophyll a (A) and total carotenoids (B) were extracted in dimethylfluoride (DMF) and quantified spectroscopically. Phycocyanin content (C) was determined by the loss of phycobiliprotein absorbance in samples after heating. Data (means±SD) are based on three independent experiments.

The amount of photosynthetic pigment content in cyanobacterial cells indicates their growth and physiological status. Figure 2 depicts the kinetics of pigment accumulation and loss in the wild type and the *pfsR* deletion mutant after exposure to iron limitation. Chlorophyll a, total carotenoid, and phycocyanin levels declined dramatically in the wild type after iron deprivation. The wild type lost almost all of the pigments after 120 hours of iron limitation, whereas the *pfsR* deletion mutant exhibited only minor changes in pigmentation. These data further verify that *pfsR* deletion alleviates iron stress in *Synechocystis* 6803.

### 77K fluorescence emission of the wild type and the *pfsR* deletion mutant during iron limitation

**Figure 3:**
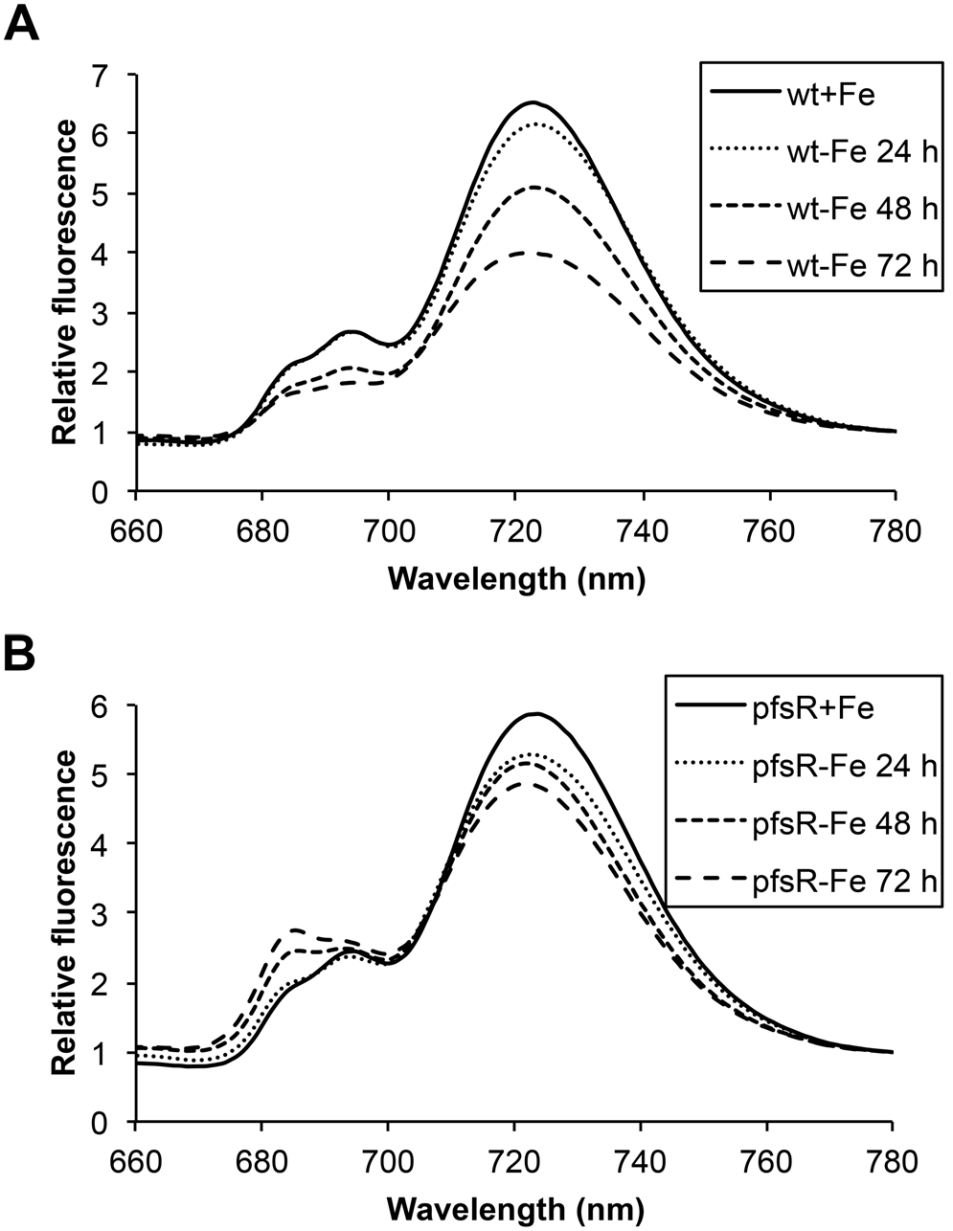
77K chlorophyll fluorescence emission spectra under iron-limiting conditions. Wild-type (A) and *pfsR* deletion mutant (B) cells were exposed to iron limitation for the indicated periods of time. The fluorescence emission spectra were recorded in liquid nitrogen at an excitation wavelength of 430 nm and normalized to the emission intensity at 780 nm. All samples were at 15 µg of chlorophyll ml^−1^. The experiment was repeated three times. Representative data are presented.

In order to monitor the changes in the photosynthetic apparatus during iron limitation, we diagnostically investigated PSI and PSII by 77K fluorescence emission spectroscopy using 430 nm light as excitation, which is preferentially absorbed by chlorophyll. As shown in Figure 3, the chlorophyll fluorescence emission spectra in the wild type and the *pfsR* deletion mutant exhibited peaks that were typical of PSII (at 685 and 695 nm), and of PSI (at 725 nm). After iron deprivation, wild-type cells exhibited a more pronounced decrease in both PSI and PSII fluorescence than did *pfsR* deletion mutant cells. This decrease correlates with the overall decrease in cellular chlorophyll content in wild-type cells as compared with *pfsR* deletion mutant cells under iron-limiting conditions. The 77K fluorescence measurements suggest that the *pfsR* deletion mutant is better able to maintain photosynthetic apparatus/complexes than the wild type under iron-limiting conditions.

Interestingly, although *Synechocystis* 6803 cells treated by 2,2′-dipyridyl underwent severe iron limitation, their chlorophyll fluorescence emission spectra did not exhibit a distinct shoulder at 685 nm as observed under iron limitation without the iron chelator. This suggests that there exist marked differences in the levels of IsiA and/or the compositions of IsiA complexes between the two different iron-limiting conditions.

### The *pfsR* deletion mutant maintains higher levels of photosystem proteins and heme than the wild type under iron-limiting conditions

**Figure 4:**
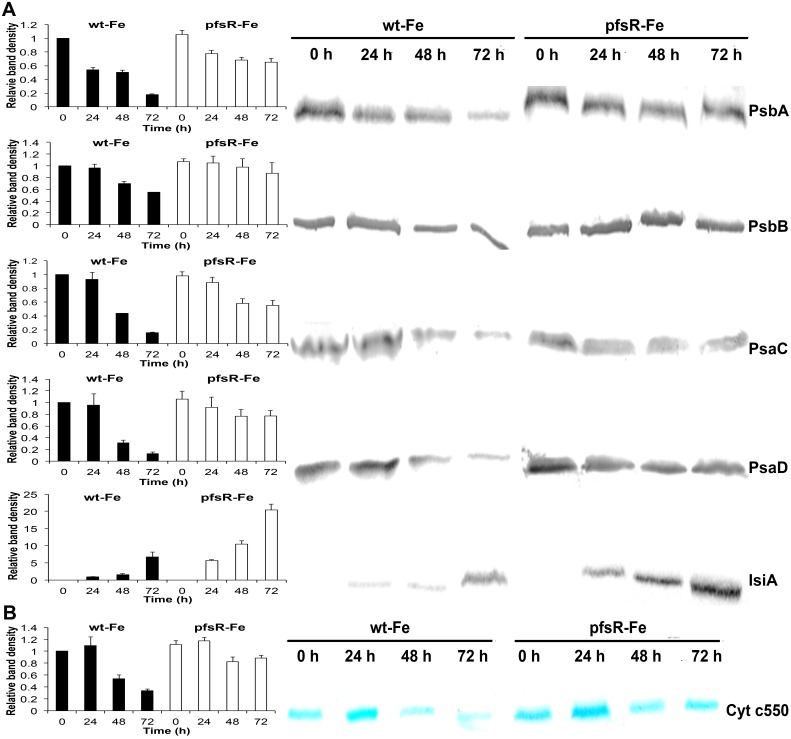
Effects of iron limitation on levels of photosystem proteins and heme. A, Immunoblot analysis of photosystem proteins. Thylakoid membrane proteins were separated by SDS-PAGE and blotted onto a nitrocellulose membrane. Proteins PsaC, PsaD, PsbA, PsbB, and IsiA were detected using specific antibodies. Each sample contained 20 µg of thylakoid membrane proteins. B, Heme staining of Cytochrome c550 using 3,3′,5,5′-tetramethylbenzidine (TMBZ). Each sample contained 20 µg of whole cell proteins. Densitometric analysis of immunoblot and heme staining results were shown on the left. Means±SD were calculated from three biological replicates. The density of bands was normalized to the values from the iron-replete wild-type cells, except for IsiA immunoblot. The density of bands in IsiA immunoblot was normalized to the values of the wild-type cells subjected to 24 hours of iron limitation.

The accumulation of photosystem proteins following iron deprivation was further examined by immunoblot analysis. Thylakoid membranes prepared from wild-type and *pfsR* deletion mutant cells that had been shifted to iron-deprived medium for various periods of time were separated on 12% SDS-PAGE gels with 6 M urea and probed using specific antibodies. As shown in Figure 4A, the wild type had markedly less PsaC and PsaD (PSI proteins) and PsbA and PsbB (PSII proteins) after exposure to iron limitation than did the *pfsR* deletion mutant. In addition, IsiA accumulated to a much higher level in the *pfsR* deletion mutant compared to the wild type under iron-limiting conditions. We also examined the heme cytochrome c_550_ content in whole cell proteins using heme staining. Similarly, iron limitation caused a more significant loss of heme in the wild type than in the *pfsR* deletion mutant (Fig. 4B). Consequently, comparing with the wild type, the *pfsR* deletion mutant exhibits stronger resistance to iron limitation.

### The *pfsR* deletion mutant exhibits relatively high photosynthetic activity and efficiency as compared with the wild type during iron limitation

We monitored photosynthetic electron transport activity under iron-limiting conditions by measuring oxygen evolution (Table 1). There were no significant differences in photosynthetic activity between the wild type and the *pfsR* deletion mutant under iron-replete conditions. However, after cells were shifted to iron-limiting conditions, the oxygen evolution activity of intact cells (as gauged by whole-chain and PSII activities) and the oxygen uptake activity of the thylakoid membranes (PSI activity) decreased more sharply in the wild type than in the *pfsR* deletion mutant.

**Table 1:** Oxygen evolution of the whole electron transport chain (whole-chain), PSII, and PSI in the wild type and the *pfsR* deletion mutant.

	Whole-Chain				PSII				PSI				
	**+Fe**	**−Fe 24 h**	**−Fe 48 h**	**−Fe 72 h**	**+Fe**	**−Fe 24 h**	**−Fe 48 h**	**−Fe 72 h**	**+Fe**	**−Fe 24 h**	**−Fe 48 h**	**−Fe 72 h**	
	µ**mol O_2_** **mg Chl^−1^h^−1^**				µ**mol O_2_** **mg Chl^−1^h^−1^**				µ**mol O_2_** **mg Chl^−1^h^−1^**				
wt	212±24	182±5	150±23	137±10	374±37	344±29	282±21	238±26	−764±53	−660±48	−376±36		−241±30
pfsR	217±28	214±18	195±15	182±20	390±43	381±30	354±29	340±39	−788±37	−721±45	−526±47		−436±32

Cells were cultured in iron-replete or iron-limiting conditions for various periods. Whole-chain-, PSII-, and PSI-mediated oxygen evolution were measured using a Clark-type oxygen electrode. Data are means±SD from three independent experiments.

**Figure 5:**
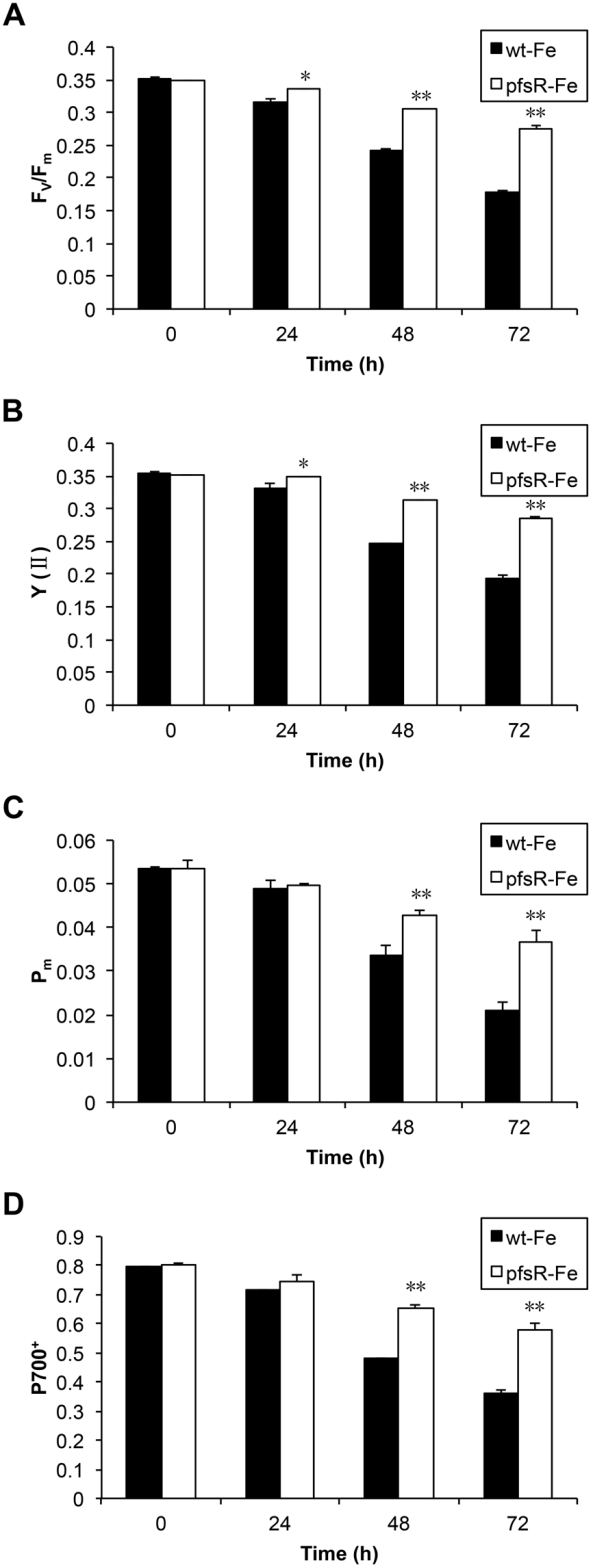
The photosynthetic activity and efficiency of wild-type and *pfsR* deletion mutant strains under iron-limiting conditions. Wild-type (black columns) and *pfsR* deletion mutant (white columns) cells were collected at different time points after iron deprivation. Changes in F_v_/F_m_ (A), Y (II) (B), P_m_ (C) and P700^+^ (D) were monitored using a modulated fluorometer at a chlorophyll concentration of 3 µg ml^−1^. The results are averages of nine measurements from three independent experiments, with error bars representing SD, a single asterisk indicating significance at P<0.05 levels, and double asterisks indicating significance at P<0.01 levels (student’s t-test).

The photochemical efficiency was also measured using room-temperature chlorophyll fluorometry. The maximum quantum yield (F_v_/F_m_) and the effective quantum yield [Y(II)] of PSII (presumably reflecting PSII activity), as well as the maximal P700 changes (P_m_) and the proportion of oxidized P700 (P700^+^) (a representative chosen for the estimation of PSI activity), all markedly decreased in response to iron deprivation in the wild type as compared with the *pfsR* deletion mutant (Fig. 5).

**Figure 6:**
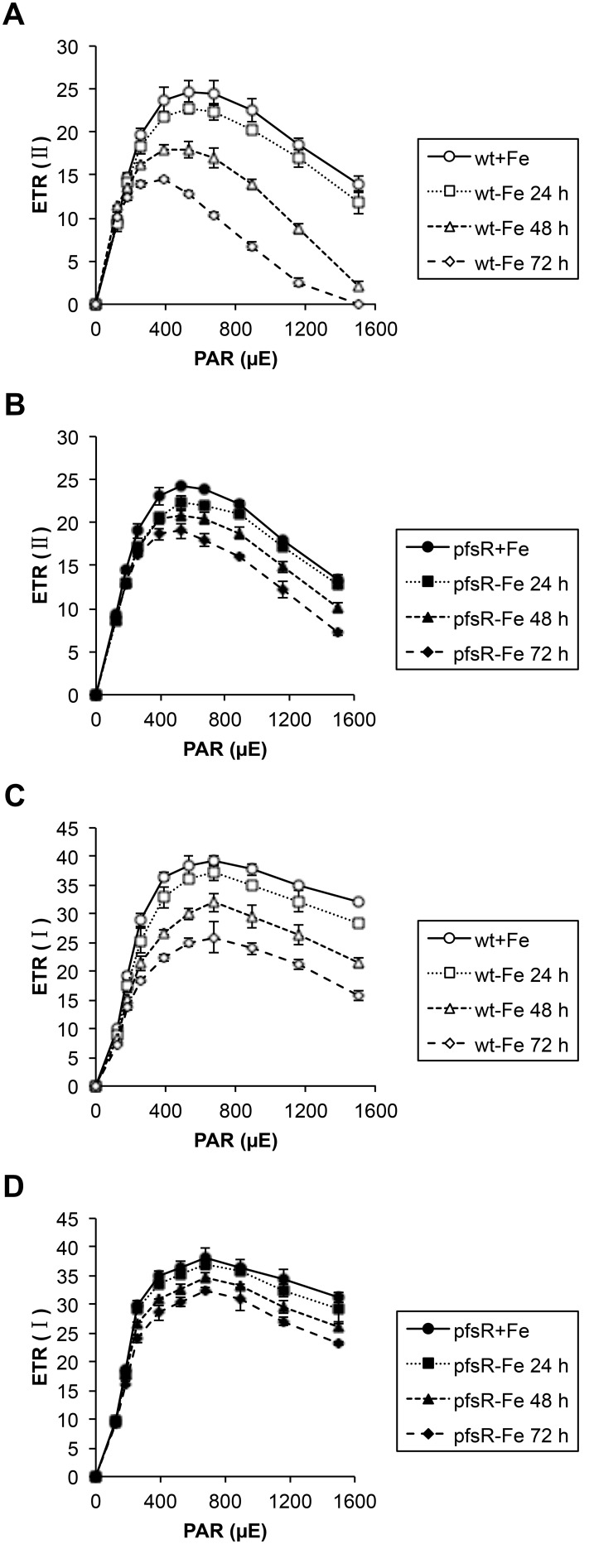
Light response curves of the wild type and the *pfsR* deletion mutant. Wild-type (A and C, open symbols) and *pfsR* deletion mutant (B and D, closed symbols) cells were exposed to iron limitation for the indicated periods of time (0 h, circles; 24 h, squares; 48 h, triangles; 72 h, diamonds). The relative electron transport rate (ETR) of PSII [ETR (II)] and PSI [ETR (I)] were assessed base on chlorophyll fluorescence measurements at stepwise increasing PAR levels. The data are means±SD of six independent measurements.

The light-intensity dependence of the relative electron transport rate (ETR) is indicative of the relative amount of electrons passing through photosystems during steady-state photosynthesis. The PSII- and PSI-mediated ETR [ETR(II) and ETR(I), respectively] in both the wild type and the *pfsR* deletion mutant were measured at stepwise increasing photosynthetically active radiation (PAR) levels. From the differences in the initial slopes of light response curves shown in Figure 6, we conclude that the relative antenna sizes of PSII and PSI in the wild type decreased more rapidly than in the *pfsR* deletion mutant after iron deprivation. These differences also explain the apparently higher maximal electron transport capacity of the iron-depleted *pfsR* deletion mutant.

Taken together, under iron-limiting conditions, the *pfsR* deletion mutant exhibits higher photosynthetic activity and efficiency than the wild type.

### *pfsR* is inducible by iron limitation and its inactivation affects pronouncedly the expression of iron stress-associated genes

**Figure 7:**
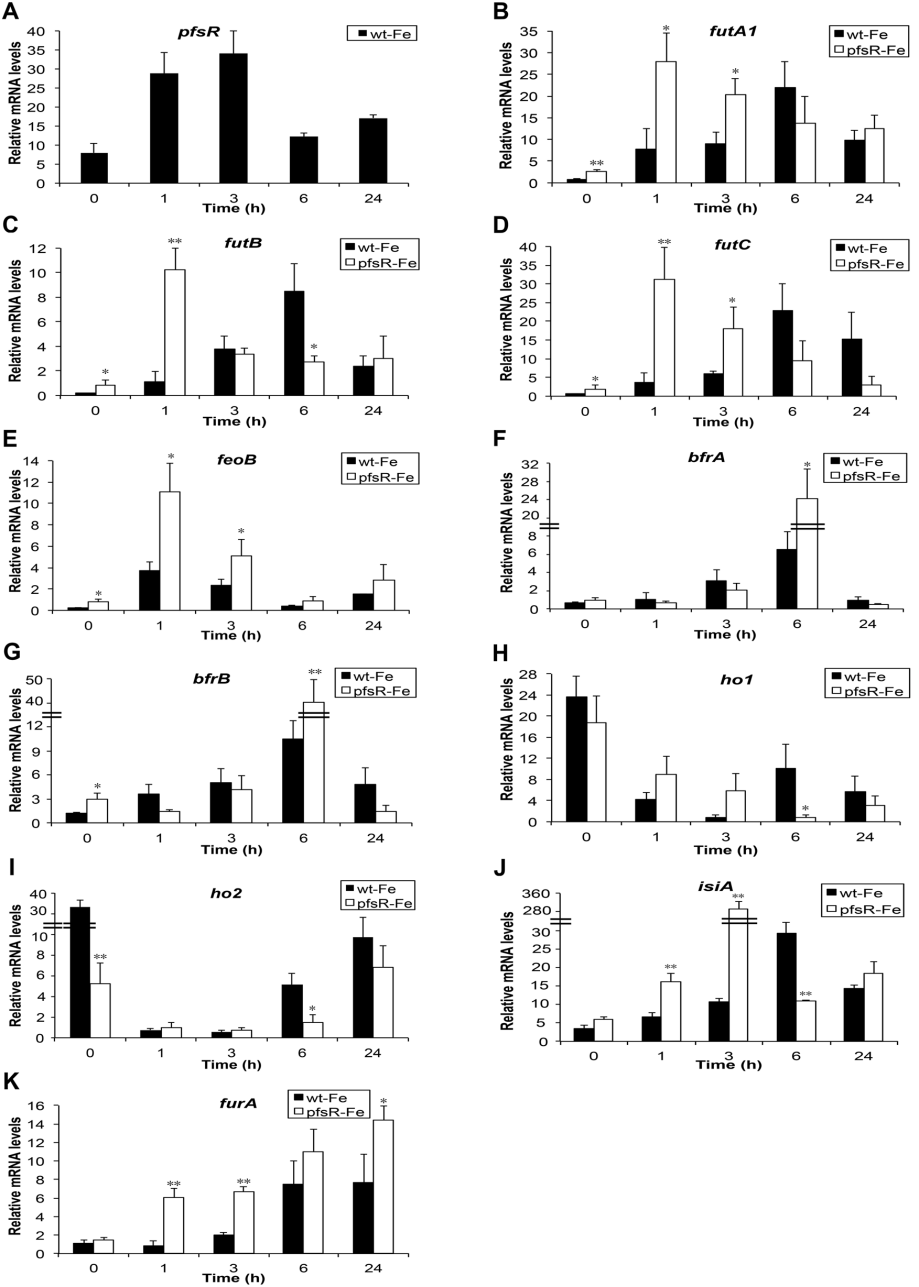
Quantitative PCR analysis of iron stress-associated gene expression following iron deprivation. The cultures were subjected to iron deprivation in the presence of 10 µM 2,2′-dipyridyl. Total RNA was isolated from the wild-type (black columns) and the *pfsR* deletion mutant (white columns) cells at the indicated time points. Gene-specific primers for *pfsR* (A), *futA1* (B), *futB* (C), *futC* (D), *feoB* (E), *bfrA* (F), *bfrB* (G), *ho1* (H), *ho2* (I), *isiA* (J), and *furA* (K) were used for quantitative PCR analysis. 16S rRNA was used as a control. The data are averages of three biological replicates, with error bars representing SD. Student’s t-test was performed for statistical analysis of significant differences between the wild type and the *pfsR* deletion mutant. A single asterisk indicates significance at P<0.05 levels, and double asterisks indicate significance at P<0.01 levels.

In cyanobacteria, transcriptional regulation is one of the most important mechanisms for modulating responses to environmental stresses. To decipher the function of PfsR, a predicted transcriptional regulator, the expression profile of *pfsR* after exposure to iron limitation, and the impacts of the *pfsR* deletion on the expression of a number of genes involved in the iron stress responses were examined by quantitative PCR (Figure 7). In wild-type cells, the *pfsR* mRNA level increased in the first hour after iron deprivation and reached peak level in three hours (with a 3.3-fold increase), after which, it declined gradually. We noticed that in previous microarray data, iron limitation led to either no change or only slight change (a 1.3-fold increase) in the transcript level of *pfsR* [3], [41]–[43]. The discrepancy among these results might be due to subtle differences in methods/conditions of iron-deprivation, sampling, and/or mRNA detection. The transcripts of ferric iron transporter encoding genes (*futA1*, *futB*, and *futC*) and ferrous iron transporter encoding gene (*feoB*) were all found to be up-regulated in the *pfsR* deletion mutant, even under iron-replete conditions. Moreover, the *fut* genes were induced immediately and peaked 1 hour after iron deprivation in the *pfsR* deletion mutant, whereas they only peaked 6 hours after iron deprivation in the wild type. The transcription of bacterioferritins, encoded by *bfrA* and *bfrB*, increased to a higher level in the *pfsR* deletion mutant than in the wild type after 6 hours of iron deprivation. In both the wild type and the *pfsR* deletion mutant, the expression of *ho1* and *ho2* genes (encoding heme oxygenases) declined in response to iron limitation. Additionally, the *ho1* and *ho2* transcript levels of the *pfsR* deletion mutant decreased more than did those of the wild type. Time course analysis of *isiA* expression revealed that the induction of *isiA* by iron limitation was much greater in the *pfsR* deletion mutant than in the wild type. In addition, the increase in the transcript level of *furA* (encoding a ferric uptake regulator) in response to iron limitation was more pronounced in the *pfsR* deletion mutant than in the wild type.

### The *pfsR* deletion mutant has higher intracellular iron content than the wild type

**Figure 8:**
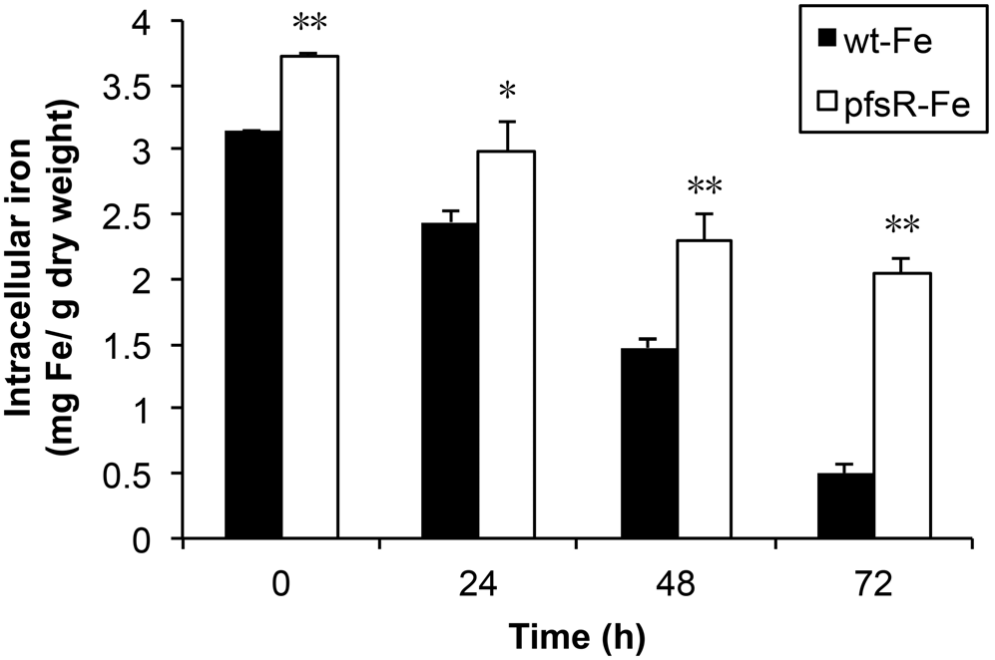
Intracellular iron content of the wild type and the *pfsR* deletion mutant. Iron content in wild-type (black columns) and *pfsR* deletion mutant (white columns) cells before (0 h) or after (24 h, 48 h, and 72 h) exposure to iron limitation was measured using atomic absorption spectroscopy. Iron content is expressed as milligram iron per gram dry cell weight. Data (means±SD) are based on two independent experiments with three atomic absorption replicates each. A single asterisk and double asterisks indicate significant difference between wild type and the *pfsR* deletion mutant at P<0.05 and P<0.01 levels, respectively (student’s t-test).

To evaluate whether the *pfsR* deletion mutant has increased iron content inside the cells, we measured intracellular iron content of the wild type and the *pfsR* deletion mutant grown in either iron-replete or iron-limiting conditions using atomic absorption spectroscopy (Fig. 8). The *pfsR* deletion mutant contained 15% more iron than the wild type under iron-replete conditions. Additionally, the intracellular iron content decreased more slowly in the *pfsR* deletion mutant than in the wild type after exposure of the cells to iron limitation. Strikingly, the *pfsR* deletion mutant contained about 240% more iron than the wild type after 72 hours of iron deprivation. Therefore, the *pfsR* deletion mutant suffers less iron deficiency than the wild type when cells are grown under iron-limiting conditions.

### PfsR specifically binds to its own promoter

**Figure 9:**
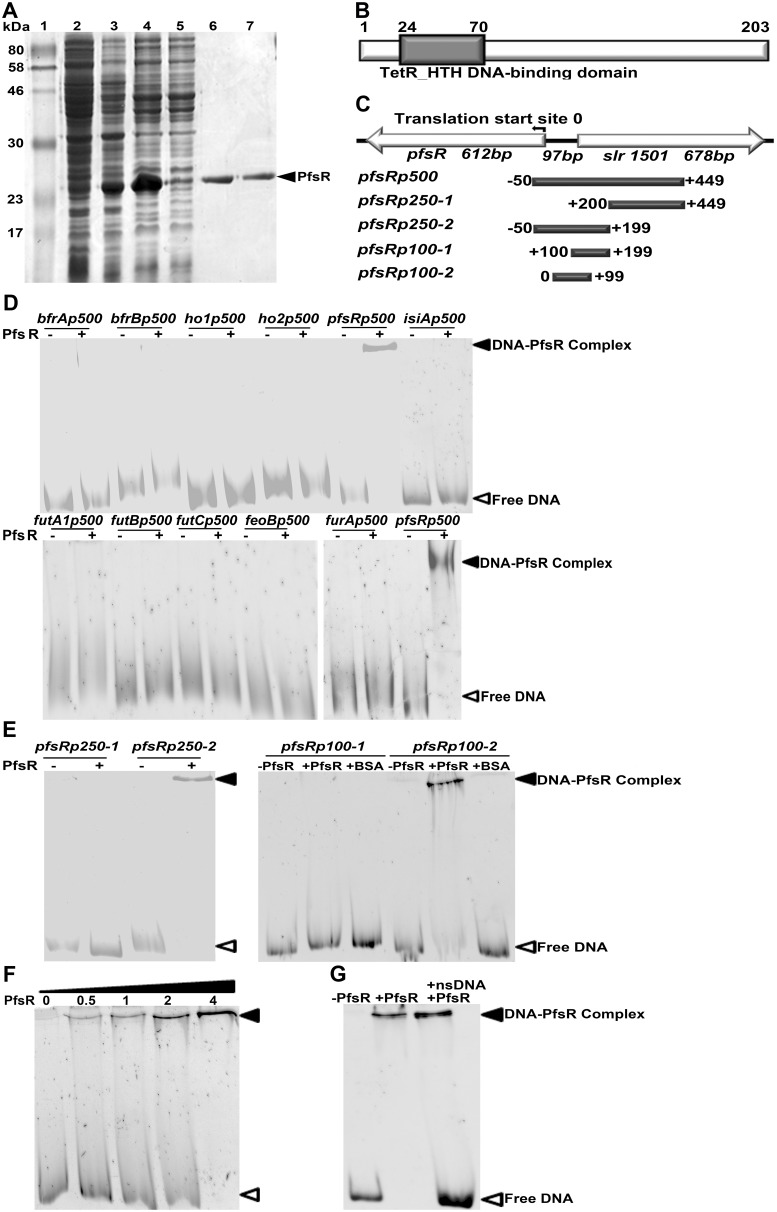
DNA-binding activity of PfsR. A, The expression and purification of recombinant *Synechocystis* 6803 PfsR. Purification was carried out using TALON spin columns. Lane 1, molecular weight standards; Lane 2, total proteins of uninduced *E. coli* cells; Lane 3, total proteins of induced *E. coli* cells; Lane 4, soluble proteins of induced *E. coli* cells; Lane 5, proteins in flow through; Lane 6 and Lane 7, eluted PfsR. The molecular mass of the standards is shown on the left. Bands were visualized using Coomassie Blue staining. B, Analysis of the domain structure of PfsR. C, Schematic representation of the genomic location of *pfsR* and the location of DNA fragments used in the EMSA. D, EMSAs for the DNA-binding activity of PfsR with the promoter DNA fragments of *futA1*, *futB*, *futC*, *feoB*, *bfrA*, *bfrB*, *ho1*, *ho2*, *pfsR, isiA*, *and furA* genes. For these assays, 0.05 µM DNA fragments were co-incubated with 2 µM purified PfsR. E, EMSA to delimit the PfsR-binding region. EMSA was performed using 0.05 µM DNA fragments containing putative PfsR-binding regions and 2 µM purified PfsR or 10 µM BSA. F, Effect of increasing PfsR concentrations on binding to its own promoter. The assay was performed with 0, 0.5 µM, 1 µM, 2 µM, and 4 µM purified PfsR, respectively, and 0.05 µM *pfsR*p100-2 DNA. G, Competitive assay for the specific DNA-binding activity of PfsR. Binding of PfsR to *pfsR*p100-2 was competed with a 20-fold excess of non-specific DNA (nsDNA). The bands corresponding to free DNA and DNA-PfsR complexes are indicated by empty and filled triangles, respectively.

To analyze the regulatory mechanism of PfsR further, we cloned *pfsR* and expressed it in *E. coli* cells. We then purified C-terminal His_6_-tagged PfsR by affinity chromatography and confirmed the identity and purity of the isolated proteins by SDS-PAGE. The His_6_-tagged PfsR, at >95% purity, had a molecular mass of about 24, 000 Daltons (24 kDa) (Fig. 9A).

The *Synechocystis* 6803 *pfsR* gene encodes a protein consisting of 203 amino acid residues. Sequence alignment analysis showed that PfsR contains a typical TetR-like N-terminal HTH DNA-binding domain (Fig. 9B). To investigate the DNA-binding activity of PfsR, we performed electrophoretic mobility shift assay (EMSA) using purified PfsR and 500-bp DNA fragments corresponding to the promoter regions of *futA1*, *futB*, *futC*, *feoB*, *bfrA*, *bfrB*, *ho1*, *ho2*, *pfsR*, *isiA,* and *furA* genes. EMSA revealed that PfsR bound to its own promoter, but not to those of the other genes (Fig. 9D). To pinpoint the exact location of the PfsR binding site, we amplified a set of DNA fragments of 250 bp and 100 bp, corresponding to the postulated *pfsR* promoter regions, by PCR (Fig. 9C). EMSA using these fragments showed that the 250-bp *pfsR*p250-2 fragment and the 100-bp *pfsR*p100-2 fragment were shifted in the presence of PfsR (Fig. 9E), indicating that PfsR bound to the region 99 bp upstream of the *pfsR* translational start site. As shown in Figure 9F, the binding of PfsR to the *pfsR*p100-2 fragment increased as the amount of protein increased. The addition of a 20-fold excess amount of non-specific DNA (containing 100 bp of the PfsR ORF region) did not inhibit shifting of the PfsR-bound *pfsR*p100-2 fragment (Fig. 9G). Together, these results indicate that PfsR specifically binds to its own promoter region.

## Discussion

Cyanobacteria are often exposed to iron deficiency due to the extremely low solubility of iron in aerobic ecosystems. In this study, we analyzed the function and regulation of PfsR, a potential regulator of iron homeostasis, under iron-limiting conditions in the model cyanobacterium *Synechocystis* 6803. The *pfsR* deletion mutant was better able to withstand iron stress than the wild type. Firstly, the *pfsR* deletion mutant grew better than the wild type under iron-limiting conditions (Fig. 1). Secondly, the *pfsR* deletion mutant accumulated chlorophyll a, carotenoid, and phycocyanin to a significantly higher level than the wild type after iron deprivation (Fig. 2). Thirdly, the *pfsR* deletion mutant maintained more PSI and PSII than the wild type after exposure to iron limitation (Fig. 3 and 4). Furthermore, the iron-limited *pfsR* deletion mutant maintained relatively high photosynthetic activity and efficiency compared to iron-limited wild type (Table 1, Fig. 5 and 6).

**Figure 10:**
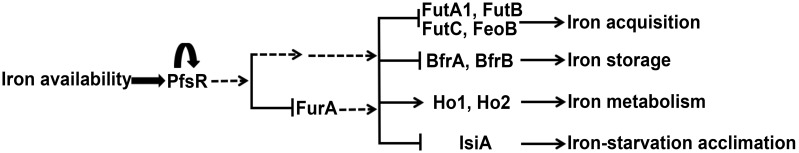
Hypothetical model for the mechanism of PfsR regulation. Auto-regulated PfsR responds to iron availability and negatively regulates the expression of genes involved in iron acquisition, storage, and starvation acclimation, but positively regulates the expression of genes related to iron release via negative control of FurA and/or the modulation of other factors. Solid arrows and bars indicate positive and negative regulation, respectively. Dashed arrows represent unknown interactions or missing steps.

Why does the *pfsR* deletion mutant survive under iron-limiting conditions? PfsR is a TetR family transcriptional regulator. The improved resistance of the *pfsR* deletion mutant to iron limitation might be conferred by the increased expression of *fut* genes, *feoB*, *bfr* genes, *isiA, furA*, and the decreased expression of *ho* genes under iron-limiting conditions (Fig. 7, 9 and 10). It might also be attributed to a relaxed control of the general responses to iron stress (which primes cells for tolerance or resistance before the onset or at the very early stage of iron limitation).

The *fut* genes encode subunits of an ATP-binding cassette (ABC)-type ferric iron transporter that is important in iron acquisition of *Synechocystis* 6803 [12], [44]. The constitutively higher expression of *fut* genes in the *pfsR* deletion mutant under iron-replete conditions and the more rapid induction of *fut* genes upon iron deprivation indicate that the *pfsR* deletion mutant is better able to absorb ferric iron from the environment. The expression of *feoB*, which encodes a ferrous iron transporter [7], was induced by iron deprivation to a greater extent in the *pfsR* deletion mutant than in the wild type. This might increase the amount of iron transported into the *pfsR* deletion mutant.

Bacterioferritins store iron when iron is sufficient, but release it under iron-limiting conditions [45], [46]. The up-regulation of bacterioferritin genes (i.e., *bfrA* and *bfrB*) in the *pfsR* deletion mutant likely promotes iron storage when iron is available, and the stored iron is subsequently used for cell growth under iron-limiting conditions. Additionally, it is reasonable to assume that the increased iron storage coincides with an increased iron uptake. The finding that the *pfsR* deletion mutant contained 15% more intracellular iron under iron-replete conditions and 240% more intracellular iron under iron-limiting conditions than the wild type supports our interpretation well (Fig. 8). Therefore, the enhanced tolerance of the *pfsR* deletion mutant to iron limitation is likely caused by better iron acquisition and iron storage.

The ferredoxin-dependent heme oxygenase catalyzes the degradation of heme to produce biliverdin IXα with the release of ferrous iron [47]–[50]. The down-regulation of both heme oxygenase genes (*ho1* and *ho2*) in the *pfsR* deletion mutant might reduce not only the demand for the iron-containing ferredoxin, but also the release of detrimental free iron that causes oxidative stress through the Fenton reaction [51], [52].

IsiA is critical for the growth of cyanobacteria under iron-limiting conditions and its induction is a hallmark of the transcriptional response to iron stress [14], [15]. IsiA can either bind to photosynthetic complexes and form supercomplexes or exist in the unbound form [9], [17]–[23]. The IsiA supercomplexes in iron-limited cells may function as an additional and efficient light-harvesting antenna, compensating for the reduction in PSI [9], [26], [27]. IsiA may also play an important role in photoprotection by dissipating excess light energy [24], [25], [28], [29]. Furthermore, as a primary chlorophyll storage protein in iron-limited cells, IsiA binds up to 50% of the total chlorophyll, thus neutralizing the potential toxic effects of free chlorophyll and allowing photosynthesis to recover rapidly when iron is resupplied [9], [10], [53], [54].

The induction of IsiA was much stronger in the *pfsR* deletion mutant than in the wild type after iron deprivation. It may seem paradoxical that the *pfsR* deletion mutant accumulated more IsiA, as the mutant suffered less iron stress than the wild type when iron was limiting. However, PfsR might be a negative regulator of IsiA, and deletion of *pfsR* would then lead to an increase in *isiA* expression, which in turn would enhance the viability of cells under iron-limiting conditions. The regulation of *isiA* expression is complex, which may involve the transcriptional control by FurA, the posttranscriptional control by the antisense RNA IsrR, and/or a presently unidentified regulatory mechanism [55], [56]. Since PfsR does not bind to *isiA* promoter directly, the regulation likely requires the participation of other factors yet to be identified.

The Fur family proteins are essential in regulating iron homeostasis of cyanobacteria [43]. It has been found that the *Synechococcus* sp. PCC 7492 FurA regulates the expression of numerous genes related to iron stress (such as *isiA* and *isiB*) [57]. The *Anabaena* sp. PCC 7120 FurA was reported to accumulate under iron-limiting conditions and act as a master regulator of iron metabolism [58]. *Synechocystis* 6803 contains three genes encoding Fur-like proteins (*sll0567*, *sll1937*, and *slr1738*), among which, the *sll0567*-ecoded FurA is most likely the functional homolog of FurA in *Synechococcus* sp. PCC 7492 and *Anabaena* sp. PCC 7120 [43], [55]. FurA was found to be involved in regulating the iron-inducible *isiAB* in *Synechocystis* 6803 [55]. Fur proteins usually use ferrous iron as a cofactor (thus can sense intracellular iron levels), bind to the specific promoter regions (Fur box) of target genes, and regulate their expression [59], [60]. The expression of Fur proteins can be self-regulated, or controlled by other stress response regulators [61]–[63], or modulated by antisense RNA [64]–[66].

In this study, we showed that inactivation of *pfsR* resulted in a much stronger induction of *furA* after iron deprivation, implying that PfsR might contribute to the control of *furA* expression. It has been reported that *feoB*, *bfr*, *ho*, and *isiA* were all potential target genes of Fur regulation [55]–[58], [67], [68], therefore, it is possible that the effect of PfsR on the expression of these genes could result from the regulation of FurA. Another possibility is that PfsR and FurA might regulate iron homeostasis through different signaling pathways, but their regulation might partially overlap.

PfsR homologues exist in other cyanobacterial strains. It is likely that these TetR family transcriptional regulators are involved in the adaptation to complex and changing environments. Indeed, one of the three TetR family regulators [encoded by *sll1392* (*pfsR*), *sll1286*, and *slr0895* (*prqR*)] in *Synechocystis* 6803, the PrqR, was found to be an auto-repressor regulating the adaptive responses to the oxidative stress [69]–[71]. Here, we demonstrated that PfsR was auto-regulated and involved in responses to iron stress.

The transcription of PfsR was activated at the early stage of iron limitation, and was then reduced gradually, suggesting an important role of PfsR at the onset of iron-stress responses. PfsR binds to its own promoter, and therefore is auto-regulated. Auto-regulation enables tight homeostatic control of protein expression in response to environmental changes [72]–[76], and thus is considered to be an ideal stress response mechanism. It is possible that deletion of *pfsR* leads to a relaxed control of certain responses to iron limitation, which may prepare the cells for iron stress before the onset or at the very early stage of iron deficiency.

We have not been able to observe any obvious defects in the fitness of the *pfsR* deletion mutant under the few growth conditions (including low light, high light, and iron limitation) we tested in the laboratory, however, we cannot rule out the possibility that the mutant carries defects in the natural habitats or under other experimental conditions yet to be tested. Apart from the *pfsR* deletion mutant, several other single gene inactivation mutants, which displayed improved stress tolerance without deleterious phenotypes, have also been reported; for example, the *Arabidopsis oxt1* mutant and the *cbf2* mutant exhibit improved tolerance to oxidative stress and freezing stress, respectively [77], [78].

The observed complexity in the expression of iron stress-associated genes suggests that multiple regulatory mechanisms might be involved in iron stress responses. While the mode of PfsR function and the cross-talks among PfsR and other iron regulators (such as FurA) invite further investigation, the finding that the inactivation of PfsR affects pronouncedly the expression of iron stress-associated genes implies a key regulatory role of PfsR in iron homeostasis. Because various stress responses might share common regulatory mechanisms and regulators [3], [30], [38], it is possible that PfsR might function in other stress responses as well. Global gene expression profiling of the *pfsR* deletion mutant under various stress conditions will provide new insights into the roles of PfsR in regulating stress responses.

The work presented here demonstrates a critical role of PfsR in regulation of iron homeostasis. As depicted in Fig. 10, we hypothesize that the improved ability of the *pfsR* deletion mutant to withstand iron limitation hinges on a mechanism that involves the auto-regulation of PfsR and the altered expression of genes involved in iron homeostasis, including those with roles in iron uptake, storage, and release.

## Supporting Information

Table S1 **List of primers used in this study.**
(DOC)
