# Supplementary material for: PfsR Is a Key Regulator of Iron Homeostasis in Synechocystis PCC 6803
Source: PLoS One. 2014 Jul 10;9(7):e101743. doi: 10.1371/journal.pone.0101743 (PMC4092027; doi:10.1371/journal.pone.0101743)
Supplement: Table S1 — List of primers used in this study. (DOC) [file pone.0101743.s001.doc]

**Table S1.** List of primers used in this study.

| Purpose | Primers | Sequence (5’-3’) | Purpose | Primers | Sequence (5’-3’) |
| --- | --- | --- | --- | --- | --- |
| RT-PCR | pfsR-RT-F | GAGGGTTTTGCCCACTTGGT | EMSA | futA1-p500-F | TGACATAATTCCGGTGGCTAAA |
|  | pfsR-RT-R | CCAAGCATTGGGCAGTTGTA |  | futA1-p500-R | GAAGGCGGTTCCAATGGACAAA |
|  | futA1-RT-F | CACAGTGGATTTGGCTCGTTT |  | futB-p500-F | AGTTCCCTTGGCATTATCTGGT |
|  | futA1-RT-R | GCGGAGATATTCCGGGACAT |  | futB-p500-R | ATTAAGCAATACCTTGGGAGGA |
|  | futB-RT-F | CAATATGCCTCCGATGAAAGACT |  | futC-p500-F | TGACCATGGTTCCCACTCTCCC |
|  | futB-RT-R | CAATTTGCACGCTCAGGAAA |  | futC-p500-R | AGAGTCTTCTATGGAAAGGCGG |
|  | futC-RT-F | GAAGCAGGCGATCGATAAATTT |  | feoB-p500-F | GTCCCAAATAGTTGCGGAGATT |
|  | futC-RT-R | AGGAAGAAGCCATGGCCATT |  | feoB-p500-R | CACATCTGGACGACTGGACTGG |
|  | feoB-RT-F | AGGACTGCTGTTGGACGGTATT |  | bfrA-p500-F | AGTACGTCCTATGCCTCAAACC |
|  | feoB-RT-R | GCCCTCTAGGATTCCCATGAC |  | bfrA-p500-R | CCCACGCAGTAGCTTATGGAGT |
|  | bfrA-RT-F | GTCCTCGCCCAACTCCATAA |  | bfrB-p500-F | AAAATTGCTAAAAAAAGGCGA |
|  | bfrA-RT-R | GCTTTTCCAGACCCCAATCTT |  | bfrB-p500-R | ATCCTCTGATGTGGAGTTTTGT |
|  | bfrB-RT-F | CGAGCGGGTTTTATTTTTGG |  | ho1-p500-F | CAGCCATTAATTTTTCCTTATC |
|  | bfrB-RT-R | GGTCAGATCATTGCCCAGGAT |  | ho1-p500-R | GTGGGATTTTTTCGTCCCTTCC |
|  | ho1-RT-F | TCCCATCCTCAGCCACATTT |  | ho2-p500-F | CTTGGCAATCCCGACTTTGGTC |
|  | ho1-RT-R | CACTTCTTGCCGCCAGTTG |  | ho2-p500-R | AGTGTGGGATTGTTGGGTACCG |
|  | ho2-RT-F | GCACGGCCATGTACGAATTT |  | isiA-p500-F | CTTGCCACATCCCCGTCGGAAG |
|  | ho2-RT-R | TCATCTAAGGGCAAGCTGTTCA |  | isiA-p500-R | ATTGCCGGCCCACCATTCGTAC |
|  | isiA-RT-F | GGTTAGCCAACGCTCACTTCT |  | furA-p500-F | ATAACCAACAAAGTTTCTGCGCTG |
|  | isiA-RT-R | GTTCGACCCGTTTGAAGTCAA |  | furA-p500-R | CCAACCACGGGCATTAAGTTCGGC |
|  | furA-RT-F | CCATCGTCTGGAAGAAGAAAGG |  | pfsR-p500-F | GCTCACGAAAACCCTCTAGGGT |
|  | furA-RT-R | CAATTCCAACTCCCGCAGAA |  | pfsR-p500-R | AGTTTGTTCTGCTTTGGTTAAC |
|  | 16SrRNA-RT-F | CTGGCGGTATGCCTAACACA |  | pfsR-p250-1-R | TGGGACGATTTGTTGGATGGTA |
|  | 16SrRNA-RT-R | TGTCCCCATTCTGAAGGTAGGT |  | pfsR-p250-2-F | AAAGCAGTGATTTAAAGGTGGT |
|  |  |  |  | pfsR-p100-1-R | GTCAACCACGCTAAATACCCGT |
| PfsR expression | pQE-pfsR-F | GTCTGCATGCTAAATCCATCCACCA |  | pfsR-p100-2-F | ATCACGACCCCTTGGGGAAAGT |
|  | pQE-pfsR-R | TGAAAGATCTACCGTAGTGATGATG |  | pfsR-p100-2-R | TCAGACAGTATGTATCTTGACA |
